# Simultaneous detection and differentiation of dengue virus serotypes 1-4, Japanese encephalitis virus, and West Nile virus by a combined reverse-transcription loop-mediated isothermal amplification assay

**DOI:** 10.1186/1743-422X-8-360

**Published:** 2011-07-21

**Authors:** Shuhua Li, Meiyu Fang, Bin Zhou, Hongxia Ni, Qiuxia Shen, Hongwei Zhang, Yifang Han, Jianhua Yin, Wenjun Chang, Guozhang Xu, Guangwen Cao

**Affiliations:** 1 Department of Epidemiology, Shanghai Key Laboratory of Medical Biodefense, Second Military Medical University, Shanghai, China; 2 Department of Comprehensive Administration, District Center for Diseases Control and Prevention of Hongkou, Shanghai, China; 3 Department of Microbiology, Center of Disease Control and Prevention of Guangzhou Military Region, Guangzhou, China; 4 Department of Emergency Medicine, First Affiliated Hospital, Second Military Medical University, Shanghai, China; 5 Department of Infectious Diseases, Center of Disease Control and Prevention of Ningbo, Ningbo, China

**Keywords:** Dengue virus, Japanese encephalitis virus, West Nile virus, Identification

## Abstract

**Background:**

Rapid identification and differentiation of mosquito-transmitted flaviviruses in acute-phase sera of patients and field-caught vector mosquitoes are important for the prediction and prevention of large-scale epidemics.

**Results:**

We developed a flexible reverse-transcription loop-mediated isothermal amplification (RT-LAMP) unit for the detection and differentiation of dengue virus serotypes 1-4 (DENV1-4), Japanese encephalitis virus (JEV), and West Nile virus (WNV). The unit efficiently amplified the viral genomes specifically at wide ranges of viral template concentrations, and exhibited similar amplification curves as monitored by a real-time PCR engine. The detection limits of the RT-LAMP unit were 100-fold higher than that of RT-PCR in 5 of the six flaviviruses. The results on specificity indicated that the six viruses in the assay had no cross-reactions with each other. By examining 66 viral strains of DENV1-4 and JEV, the unit identified the viruses with 100% accuracy and did not cross-react with influenza viruses and hantaviruses. By screening a panel of specimens containing sera of 168 patients and 279 pools of field-caught blood sucked mosquitoes, results showed that this unit is high feasible in clinical settings and epidemiologic field, and it obtained results 100% correlated with real-time RT-PCR.

**Conclusions:**

The RT-LAMP unit developed in this study is able to quickly detect and accurately differentiate the six kinds of flaviviruses, which makes it extremely feasible for screening these viruses in acute-phase sera of the patients and in vector mosquitoes without the need of high-precision instruments.

## Background

Dengue virus (DENV), Japanese encephalitis virus (JEV), and West Nile virus (WNV) belong to the members of the genus *Flavivirus *of the family *Flaviviridae*. DENV has four distinct serotypes, DENV1-4, is maintained in a human-mosquito transmission cycle involving primarily *Aedes aegypti *and *Aedes albopictus*, and results in various clinical manifestations ranging from asymptomatic to dengue shock syndrome. Global prevalence of dengue has grown dramatically in the recent decades [1]. JEV is transmitted among birds, pigs, and other domestic animals and human beings by *Culex *species mosquitoes. Humans are the dead-end hosts. JEV infection can cause severe central nervous system disorders including encephalitis, with an estimated 50,000 cases and 10,000 deaths annually in the world. Approximately 3.0 billion people live in the areas affected by JEV [2]. WNV circulates in natural transmission cycles involving primarily *Culex *mosquitoes and birds. Humans and other mammals are incidental hosts. In most cases, infection with WNV causes a self-limited febrile illness. However, this infection may also lead to encephalitis. WNV epidemic in North America is a major public health concern [3]. In most circumstance, these viruses appear transiently in acute sera after onset of the diseases and transmit quickly by mosquito bites. This has necessitated the development of rapid detection methods for these viruses in acute sera of the patients before admission to hospital and in vector mosquitoes for the prediction and prevention of large-scale epidemics.

Routine laboratory diagnosis of flavivirus infection is based on virus isolation and characterization, the detection of virus-specific antigens or antibodies, and the detection of genomic sequences by nucleic acid amplification techniques [4-6]. Virus isolation methods require time-consuming culturing. Although the detection of antibodies is widely used, the antibodies might be cross-reactive with other closely related flaviviruses [7,8]. PCR-based techniques have gradually been accepted as new standards over virus isolation for the rapid identification of DENV, JEV, and WNV. The amplification methods have the intrinsic disadvantage of requiring either a high-precision instrument for amplification or an elaborate complicated method for the detection of amplified products, which restricts its applications to the laboratories without high-precision instruments and in epidemiological field.

The loop-mediated isothermal amplification (LAMP) is a novel method based on the principle of strand displacement reaction and the stem-loop structure that efficiently amplifies the target with high specificity and sensitivity [9,10]. Reverse-transcription (RT) LAMP has been developed for the identification of DENV, JEV, and WNV, respectively [11-13]. However, the individual RT-LAMP method has not been combined and applied for the identification of the flaviviruses in acute-phase sera and vector mosquitoes. Furthermore, kinetics of the target DNA amplification and cross-reactivity within the flaviviruses or with other RNA viruses have not been fully evaluated. In this study, we developed a RT-LAMP unit for the detection and differentiation of DENV1-4, JEV, and WNV. This method can detect and differentiate the flaviviruses efficiently in acute sera of the patients and the field-caught mosquitoes.

## Materials and methods

### Viral strains and viral fragment

Four DENV reference strains (DENV1, Hawaii; DENV2, New Guinea; DENV3, H87; and DENV4, H241) and 52 DENV strains isolated in Guangdong and Zhejiang [14] of China were included in this study. Fourteen JEV strains that had been isolated in China during 1999-2008 were kindly provided by Dr. Guodong Liang (Viral Research Institute, Chinese Center for Diseases Control and Prevention, Beijing, China) and Dr. Zhaokui Zhu (Municipal Center for Diseases Control and Prevention of Shanghai, Shanghai, China). DENV and JEV strains were propagated in brains of baby mice in a biosafety laboratory. A DNA fragment of WNV at the position from nt.1021 to nt.1240 of the reference strain NY99 [GenBank: AF196835.2] was synthesized (GeneCore BioTechnologies, Shanghai, China), and then cloned into Sma I digested pUC57 plasmid (GeneCore) according to the manufacturer' specifications, confirmed by DNA sequencing. Control viruses included 12 strains of seasonal influenza viruses and 12 strains of hantaviruses recently isolated in China.

### Field-caught mosquitoes and sera of the patients

Blood sucked female mosquitoes were caught in high-risk areas where dengue and Japanese encephalitis activities were defined. *Culex tritaeniorhynchus *were caught in July 2010 around piggeries where sporadic cases with Japanese encephalitis were found in Zhejiang and Shanghai. *Aedes aegypti *were caught in residential buildings from June to August 2008 during dengue fever (DENV1) outbreak in Guangdong. *Aedes albopictus *were caught in residential buildings from July to September 2009 during dengue fever (DENV3) outbreak in Zhejiang. The mosquitoes were transported on dry ice and pooled by species into groups of up to 50 individuals. Sera of 75 sporadic cases with Japanese encephalitis diagnosed between 2007 and 2010 were obtained from our sentinel hospitals in Zhejiang and Shanghai. Sera of 93 patients with dengue fever during the disease outbreaks in 2004 (DENV1) and in 2009 (DENV3) were obtained from our sentinel hospitals in Zhejiang. The serum samples were mostly collected within 7 days after the onset of the diseases and frozen in -80°C. Sera of healthy blood donors were obtained from the physical examination center, the first affiliated hospital of this university. The study protocol using human sera conformed to the ethical guidelines of the 1975 Declaration of Helsinki, and was approved by the Institutional Review Board of this university.

### RNA extraction, reverse transcription, and quantification

The mosquito pools were homogenized by using a sample disruptor (Tissue lyser II, Qiagen, Hilden, Germany) in a total volume of 200 μL and then centrifuged at 14,000 × g for 5 min. Total RNAs were extracted from 200 μL mouse cerebral suspension, 140 μL mosquito suspension and 200 μL patients' sera using the QIAamp viral RNA mini kit (Qiagen, Hilden, Germany) according to the manufacturer's instructions, respectively. Total RNAs and WNV DNA were quantified using the NanoDrop 1000 spectrophotometer (Thermo, Wilmington, DE). Real-time TaqMan PCR method was used to determine viral concentration of Hawaii DENV-1, with forward primer 5'-TGAACATAATGAACAGGAGGAAAAGAT-3', reverse primer 5'-CCCTCGGGTGGTCAGATG-3' and TaqMan probe 5'-(Fam)TGTTACCATGCTCTTCATGCTGCTGCC(TAMRA)-3'. The primers and probe were designed using Primer Premier 5.0 software and synthesized (GeneCore). The standard controls with given viral concentrations (GeneCore) were used to determine viral concentration of DENV1. Viral RNA was reversely transcribed in 20 μL reaction volume using random hexamer oligonucleotides with the reverse transcription system (Promega, Madison, WI). A 20 mL reaction mixture contained 2 mL cDNA, 10 mL 2 × Premix Ex TaqMan™(TaKaRa, Dalian, China), and 0.4 mL of 10 mM PCR primers of each, 0.8 mL of 10 mM TaqMan probe and 6.4 mL ddH_2_O. A LightCycler™480 real-time PCR thermoblock (Roche, Basel, Switzerland) were programmed to denature the samples for 10 s at 95ºC, followed by 40 cycles of 95ºC for 10 s, 60ºC for 20 s, and 72ºC for 30 s.

### Optimization of RT-LAMP conditions for simultaneous amplification

The alignment of the reference sequences downloaded from GenBank indicated the reserved regions in the genomes of the six viruses. The forward outer primer (F3), backward outer primer (B3), forward inner primer (FIP), backward inner primer (BIP), forward loop primer (FLP), and backward loop primer (BLP) for the amplification of DENV1-4, JEV, and WNV were designed using the Primer Explorer version 3 https://primerexplorer.jp/lamp3.0.0/index.html. After comparison, the best primer sets to amplify these viruses simultaneously were the published ones [11-13]. We optimized the components of the reaction mixture to realize efficient amplification of the six viruses under the same condition. RT-LAMP reaction was carried out in a total of 25 μL reaction volume containing 7 μL primer mixture, 16 μL reaction mixture and 2.0 μL viral template. The primer mixture contained 2 μL 20 μM FIP, 2 μL 20 μM BIP, 0.5 μL 10 μM F3, 0.5 μL 10 μM B3, 1.0 μL 20 μM FLP and 1.0 μL 20 μM BLP. The reaction mixture contained 2.5 μL 10×Buffer (200 mM Tris-HCl, pH8.8, 100 mM KCl, 100 mM (NH4)_2_SO_4_, 20 mM MgSO_4_, and 1% Triton X-100), 2.0 μL *Bst *DNA polymerase (New England Biolabs, Ipswich, MA, 8 U/μL), 3.5 μL 10 mM deoxynucleoside triphosphate (Promega), 0.5 μL AMV reversed transcriptase (5 U/μL, Promega), 1.5 μL 100 mM MgSO_4_, 4.0 μL 5 M betaine, 1.0 μL RNase-free ddH_2_O, and 1.0 μL fluorescent detection reagent (Eiken Chemical Co., Ltd, Tokyo, Japan). Viral RNAs (2.0 μL) served as templates. For LAMP reactions, 0.5 μL AMV and 2.0 μL RNA in RT-LAMP were replaced with 0.5 μL ddH_2_O and 2.0 μL DNA template, respectively. The reaction was carried out at 63°C for 15-30 min and then terminated at 80°C for 2 min. Both of viral RNAs and corresponding cDNAs were evaluated as positive controls. Positive and blank controls were included for the examination of each virus.

### Development of a combined RT-LAMP unit

For simultaneous detection of DENV1-4, JEV, and WNV, we developed a flexible RT-LAMP unit composed of six rows and each row had four 0.2 ml MicroAmp reaction tubes with cap (Applied Biosystems, Foster City, CA). Each tube contained 16 μL reaction mixture and 7 μL primer mixture of a given virus. Each row containing a positive control tube (viral RNA or cDNA), two testing tubes and a blank control tube examined one of the six viruses.

### Sensitivity and specificity of the RT-LAMP unit

To evaluate the sensitivity, viral RNAs (DENV1-4 and JEV) were 10-fold serially diluted and used as templates for RT-LAMP and one-step RT-PCR. WNV DNA was 10-fold serially diluted and used as template for LAMP and routine PCR. To evaluate the specificity, a 10 μL aliquot of each RT-LAMP product was digested with the *Ban *II (Promega) for DENV1-4, the *Hha *I (Promega) for JEV, and the *Alu *I (Promega) for WNV. The amplification products and the corresponding digests were analyzed by electrophoresis on a 3% agarose gel, stained with ethidium bromide, and visualized with a Molecular Imager^® ^Gel Doc™ XR System (Bio-Rad, Hercules, CA). The authenticity of the amplified products was also verified by nucleotide sequencing of digested products. Cross-reactivity was evaluated within the six flaviviruses or with influenza viruses and hantaviruses.

### DNA amplification monitored by real-time PCR engine

Real-time monitoring of DNA amplification in LAMP reactions were performed using the LightCycler™480 real-time PCR Thermoblock by adding 1 μL 20 × SYBR Green I (Qiagen) to replace 1 μL fluorescent detection reagent in 25 μL LAMP reactions. The condition was 90 cycles at 63ºC for 40 s. Fluorescence signal of SYBR Green I at wavelength ranging from 483 nm to 533 nm was automatically recorded during the amplification.

### One-step RT-PCR

Each of viral samples was amplified in a 50 μL reaction containing 10 μL 5× reaction buffer, 2 μL 25 mM MgSO_4_, 1 μL 10 mM dNTP Mix, 0.5 μL each of 50 μM primer F3 and B3 mixture, 1 μL 5 U/μL AMA, 1 μL 5 U/μL *Tfl *DNA polymerase, 2 μL viral RNA, and RNase-free H_2_O. Amplification by RT-PCR was performed using an autorisierter thermocycler (Eppendorf AG, Hamburg, Germany). The thermal profile consisted of a 45 min reverse transcription step at 48°C and 2 min of Taq polymerase activation at 94°C, followed by 35 cycles of PCR (94°C for 30 s, annealing temperature 55°C for 60 s, and 68°C for 2 min).

### Real-time RT-PCR

Viral RNA was reversely transcribed into cDNA as described above. Real-time PCR was performed in a 20 μL reaction containing 2 μL cDNA templates, 10 μL 2× SYBR^® ^Premix Ex Taq™(TaKaRa), 2 μL 10 μM F3, 2 μL 10 μM B3, and nuclease-free ddH_2_O. The LightCycler™480 Thermoblock was programmed to denature the samples for 10 s at 95ºC, followed by 40 cycles of 95ºC for 10 s, 56ºC for 20 s, and 72ºC for 30 s.

## Results

### Specificity and sensitivity of the RT-LAMP unit

**Figure 1:**
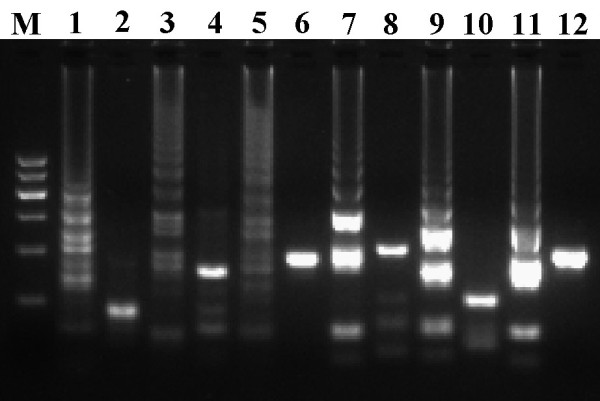
**Examination of DENV1-4, JEV, and WNV by RT-LAMP**. M, DNA ladder; 1-2, DENV1 products and digested with the *Ban II*; 3-4, DENV2 products and digested with the *Ban II*; 5-6, DENV3 products and digested with the *Ban II*; 7-8, DENV4 products and digested with the *Ban II*; 9-10, JEV products and digested with the *Hha I*; 11-12, WNV products and digested with the *Alu *I.

Under the amplification condition, DENV1-4, JEV, and WNV were successfully amplified in the RT-LAMP unit and observed as ladder-like patterns on the gel (Figure 1). The resultant digested products of 109 bp, 132 bp, 172 bp, 186 bp, 121 bp, and 175 bp were in agreement with the predicted sizes of DENV1-4, JEV, and WNV, respectively. Viral RNAs and corresponding cDNAs as templates had the same efficiency in RT-LAMP reaction. DNA sequencing of the digested products confirmed the specific amplification (data not shown). The reaction caused changes of turbidity and color. The color change from orange to green was visible under the natural light. Through the cross-reaction tests, we demonstrated that the six primer sets were specific for the amplification of the corresponding viruses (data not shown).

**Figure 2:**
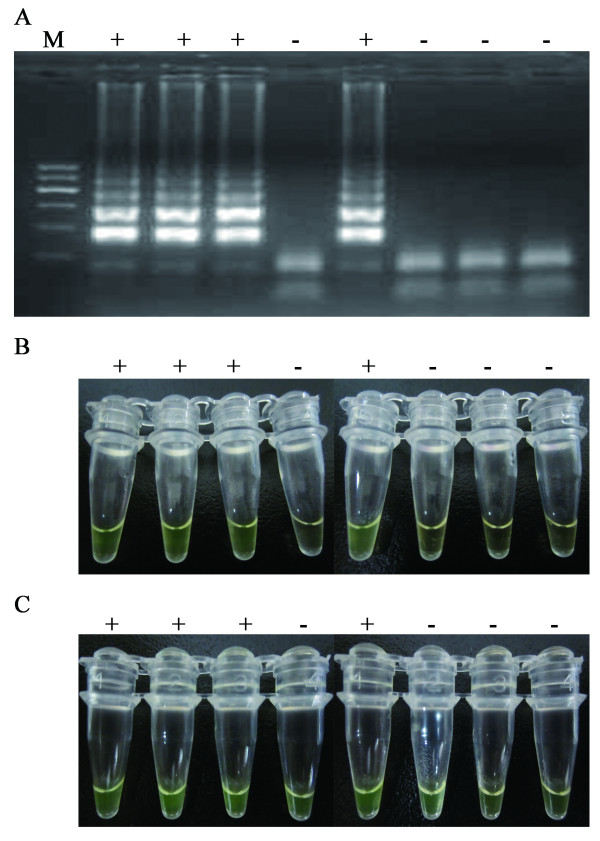
**Dynamics of electrophoresis feature and color change during the reaction of RT-LAMP for the amplification of DENV1 RNA**. A, electrophoresis feature of DNA amplicons at 30 min after the reaction started. B, color change in the row at 20 min after the reaction started. C, color change in the row at 30 min after the reaction started.

The color change in the reactions with positive controls and positive samples was evident at 15 min after RT-LAMP initiation. However, when the reactions lasted for more than 30 min, the reactions of blank controls also changed their color but do not have specific DNA amplification, as shown in Figure 2. The color change in the reactions lasted for more than 30 min could not accurately reflex the amplification results. Therefore, all of subsequent RT-LAMP results were documented within 20 min following the reactions initiation.

**Figure 3:**
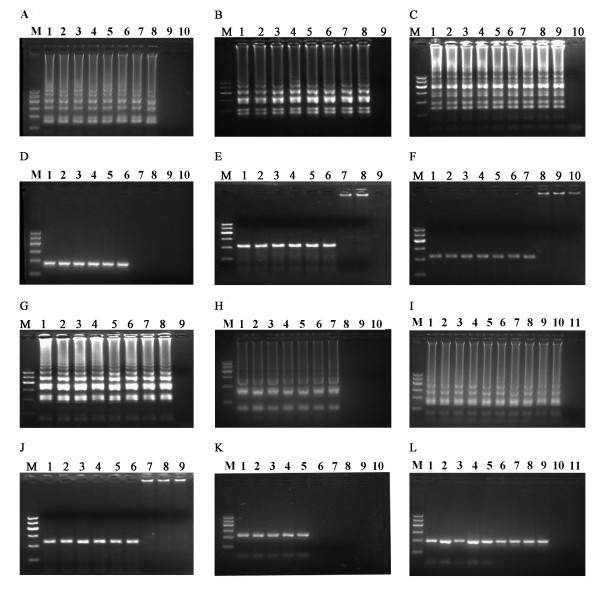
**Comparative sensitivity of RT-LAMP (LAMP for WNV) and one-step RT-PCR (PCR for WNV)**. A-C, G-I, the sensitivities of RT-LAMP assays for the amplification of DENV1, DENV2, DENV3, DENV4, JEV, and WNV, respectively. M, 100-bp DNA ladder; 1 to 10, serial 10-fold dilution of RNA templates from 10^0^. D-F, J-L, the sensitivities of RT-PCR for amplification of DENV-1, DENV2, DENV3, DENV4, JEV, and WNV with the same dilutions as A-C, G-I, respectively.

The concentrations of total RNAs extracted from the laboratory-preserved strains were 70-482 ng/µL for DENV1-4 and 75-77 ng/µL for JEV. The concentration for the synthesized WNV plasmid was 297.5 ng/mL. With the use of TaqMan PCR technique, DENV1 cDNA concentration was determined as 6.6 × 10^7 ^copies/μL. DENV1 RNA was 10-fold serially diluted and added into RT-LAMP reactions. RT-LAMP actively amplified DENV1 template even at 10^7^-fold dilution. As shown in Figure 3, RT-LAMP was 100-fold more sensitive than one-step RT-PCR for the amplification of DENV1-4 and JEV, and 10-fold more sensitive than one-step PCR for the amplification of WNV DNA.

### Dynamics of target DNA amplification

**Figure 4:**
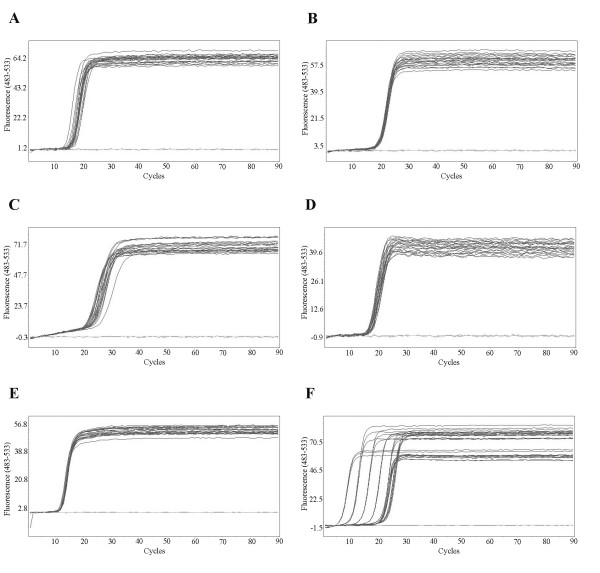
**The kinetics of RT-LAMP amplification of DENV1-4, JEV, and WNV templates with serial 10-fold dilutions**. The *x *axis depicts the cycles of amplification, and the *y *axis shows DNA concentrations in terms of the fluorescence values at 483-533 nm wavelength. A, DENV1 diluted from 10^0^- to 10^6^- folds; B, DENV2 diluted from 10^0^- to 10^6^- folds; C, DENV3 diluted from 10^0^- to 10^6^- folds; D, DENV4 diluted from 10^0^- to 10^6^- folds; E, JEV diluted from 10^0^- to 10^5^- folds; F, WNV diluted from 10^0^- to 10^9^- folds.

Ten-fold serially diluted viral genomic RNAs were added into RT-LAMP reactions, respectively. RT-LAMP efficiently amplified viral genomes at the dilution folds from 10^0 ^to 10^7 ^for DENV1-4 and from 10^0 ^to 10^6 ^for JEV, exhibiting the similar amplification curves (Figure 4). However, the amplification curve of WNV was different from those of other cDNAs at the 10^0^- to 10^6^- fold dilution, possibly because WNV DNA concentrations at 10^6^-fold dilution were still high. RT-LAMP reactions amplified the targets faster than the real-time RT-PCR. The initiation of amplification (Ct value) of LAMP was 10 to 15 cycle (7 to 10 min), while that of real-time RT-PCR was 20 to 25 cycle (20 to 25 min). LAMP needed 15-20 min, whereas real-time PCR required 40 min approximately, to reach plateau phases.

### Evaluation of the RT-LAMP unit using viral strains, acute sera, and field-caught mosquitoes

Seventeen strains of DENV1, 13 strains of DENV2, 10 strains of DENV3, 12 strains of DENV4, 14 strains of JEV, 12 strains of influenza viruses, and 12 strains of hantaviruses were examined using the RT-LAMP unit, real-time RT-PCR, and one-step RT-PCR, respectively. As shown in Table 1, the RT-LAMP unit and real-time RT-PCR identified the 66 samples with 100.0% accuracy, whereas one-step RT-PCR correctly detected 87.9% of the samples. No cross-reaction was found among DENV1-4 and JEV. The unit was unable to detect genomic RNAs of influenza viruses and hantaviruses.

**Table 1:** Identification of DENV 1-4 and JEV by the RT-LAMP unit, real-time RT-PCR, and one-step RT-PCR using viral templates at 10^5^-fold dilution

Viruses	No. of strains	No. of strains correctly identified with primer sets of DENV 1-4 and JEV, respectively			
		
		One step RT-PCR	Real-time RT-PCR	RT-LAMP unit	Cross-reaction of RT-LAMP *
DENV-1	17	15	17	17	0
DENV-2	13	11	13	13	0
DENV-3	10	9	10	10	0
DENV-4	12	11	12	12	0
JEV	14	12	14	14	0
Influenza viruses**	12	0	0	0	nd
Hantaviruses**	12	0	0	0	nd

*A given primer set in the RT-LAMP unit was used to amplify other viral templates. ** These viruses were amplified with the primer sets of the five flaviviruses, individually. nd, not done. DENV, dengue virus; JEV, Japanese encephalitis virus; RT-PCR, reverse-transcription polymerase chain reaction; RT-LAMP, reverse-transcription loop-mediated isothermal amplification.

The sera of 68 patients diagnosed with DENV1 infection (Zhejiang, 2004), 25 patients with DENV3 infection (Zhejiang, 2009) and 75 patients with JEV infection (Zhejiang and Shanghai, 2007-2010) were examined using the RT-LAMP unit, real-time RT-PCR, and one-step RT-PCR, respectively. With the use of the RT-LAMP unit or real-time RT-PCR, 7 of the 68 samples (Zhejiang, 2004) were positive for DENV1, 4 of the 25 samples (Guangdong, 2009) were positive for DENV3, 5 of the 75 samples (Zhejiang and Shanghai, 2007-2010) were positive for JEV, respectively. With the use of one-step RT-PCR, 5 of the 68 samples were positive for DENV1, 2 of the 25 samples were positive for DNEV3, and only one of the 75 samples was positive for JEV, respectively. None of 15 healthy blood donors was positive for these viruses, as examined by the three methods. We examined pools of blood sucked female mosquitoes using the RT-LAMP unit, real-time RT-PCR, and one-step RT-PCR, respectively. WNV was not found neither in the patients' sera nor in the blood sucked mosquitoes. The RT-LAMP unit was equal to real-time RT-PCR in identifying and differentiating the flaviviruses in the blood sucked female mosquitoes, as shown in Table 2. The RT-LAMP unit's amplicons of the sera and mosquitoes were subjected for DNA sequencing, and the results confirmed the specific amplification (data not shown).

**Table 2:** Viral infection in the blood sucked female mosquitoes by using the RT-LAMP unit, real-time RT-PCR, and one-step RT-PCR

Positive pools/pools of mosquitoes examined (viruses)			
	***Culex tritaeniorhynchus***,	***Aedes aegypti***,	***Aedes albopictus***,

RT-LAMP unit	18/135 (JEV)	3/98(DENV1, 3)	1/46 (DENV1)
Real-time RT-PCR	18/135 (JEV)	3/98 (DENV1, 3)	1/46 (DENV1)
One-step RT-PCR	10/135 (JEV)	1/98 (DENV1)	0/46

DENV, dengue virus; JEV, Japanese encephalitis virus; RT-PCR, reverse-transcription polymerase chain reaction; RT-LAMP, reverse-transcription loop-mediated isothermal amplification.

## Discussion

We developed a flexible RT-LAMP unit for the simultaneous identification and quick differentiation of DENV1-4, JEV, and WNV, the major flaviviruses transmitted via mosquito bites. By examining 66 laboratory-preserved strains of flaviviruses, this unit was as sensitive as a real-time RT-PCR assay and the five flaviviruses (DENV1-4 and JEV) in the assay did not cross-react with each other or with hantavirus and influenza viruses (Table 1). The RT-LAMP unit was also equal to real-time RT-PCR in identifying and differentiating the flaviviruses either in acute-phase sera or in the field-caught blood-sucked mosquitoes. Real-time RT-PCR is the most reliable method for the detection of RNA genomes of the flaviviruses [15-17], but it needs the high-precision instrument. RT-LAMP has been proven to be able to detect other viruses of interest in vector mosquitoes and clinical samples with high sensitivity and specificity [18,19], however, combined RT-LAMP for the detection of the flavivirues in vector mosquitoes and acute sera of the patients has not been reported. In this study, we demonstrated that the RT-LAMP unit was feasible in screening the flaviviruses in acute sera and vector mosquitoes without the need for high-precision instruments.

The reported methods for the detection of LAMP results are real-time monitoring of turbidity (the optical density at 400 nm) with a Loopamp real-time turbidimeter, "ladder-like feature" by agarose gel electrophoresis and color change from orange to green caused by fluorescent detection reagent [9,11-13,19]. However, real-time monitoring of DNA amplification of RT-LAMP has rarely been reported. In this study, we examined the kinetics of DNA amplification during RT-LAMP reactions using a real-time PCR engine. It was found that RT-LAMP efficiently amplified the targets at wide ranges of viral concentrations, and exhibited similar amplification curves (Figure 3). The concentration of the targets do not significantly affect specific amplification of RT-LAMP, indicating that the RT-LAMP unit is quite useful for the identification of these flaviviruses in the patients and mosquito vectors with unknown viral loads.

We observed that the color change from orange to green in the positive control was evident in first 15-20 min, while the reactions with blank controls and negative samples also changed the color from orange to green if the unit were incubated at 63ºC for more than 30 min (Figure 2). This result is not consistent with the published one that a 65ºC incubation for 60 min yielded the best result [19]. The reason is unknown. This difference is probably due to distinct reaction mixtures used in the two studies. A self-made reaction mixture with a known concentration of each component was used in this study, while the patented Loopamp RNA amplification kit was used in the published one [19]. Based on this observation, we suggest that the time point to determine the results of the RT-LAMP unit is important. In addition, determination of the result with naked eyes might not be accurate. Thus, the results of RT-LAMP unit in quickly screening the six flaviviruses from epidemiologic fields also need further confirmation in laboratories.

This study shows that RT-LAMP reaction is able to amplify specific target with several molecules. However, high sensitivity of the RT-LAMP unit also indicates the immediate necessities to prevent cross contamination because minor contamination with other targets might be enough to cause a wrong amplification. Cross contamination is one of the most important concerns for the development of combined RT-LAMP unit. We experienced failures in specific RT-LAMP amplification due to cross contamination. From our experiences, the points to block the cross contamination are (1) electrophoresis and sample handling should be done in separated lab rooms by different people; (2) changing lab coat, mask, and gloves when handling different samples; (3) keeping the cap of the tubes tightly closed during RT-LAMP reaction; and (4) using pipette tips with filter when the same pipetman was used to move different viral targets.

## Conclusion

The RT-LAMP unit developed in this study allows a simultaneous detection and rapid differentiation of DENV serotype1-4, JEV, and WNV in a wide range of template concentration. The RT-LAMP unit is extremely useful for screening the flaviviruses in acute-phase sera from the patients and in field-caught blood sucked mosquitoes.

## List of abbreviations

DENV: dengue virus; JEV:Japanese encephalitis virus; WNV:West Nile virus; RT-LAMP: reverse-transcription loop-mediated isothermal amplification.

## Competing interests

The authors declare that they have no competing interests.

## Authors' contributions

GC designed this study and wrote the paper; SL, MF, BZ, and HN carried out this study; QS, HZ, and JY collected the clinical samples and analyzed the data; YH, WC, HZ, and GX revised the manuscript critically. All of the authors read and approved the final version of this manuscript.
